# Deletion of Exon 20 of the Familial Dysautonomia Gene Ikbkap in Mice Causes Developmental Delay, Cardiovascular Defects, and Early Embryonic Lethality

**DOI:** 10.1371/journal.pone.0027015

**Published:** 2011-10-28

**Authors:** Paula Dietrich, Junming Yue, Shuyu E., Ioannis Dragatsis

**Affiliations:** Department of Physiology, College of Medicine, The University of Tennessee, Health Science Center, Memphis, Tennessee, United States of America; Instituto Nacional de Câncer, Brazil

## Abstract

Familial Dysautonomia (FD) is an autosomal recessive disorder that affects 1/3,600 live births in the Ashkenazi Jewish population, and leads to death before the age of 40. The disease is characterized by abnormal development and progressive degeneration of the sensory and autonomic nervous system. A single base pair substitution in intron 20 of the Ikbkap gene accounts for 98% of FD cases, and results in the expression of low levels of the full-length mRNA with simultaneous expression of an aberrantly spliced mRNA in which exon 20 is missing. To date, there is no animal model for the disease, and the essential cellular functions of IKAP - the protein encoded by Ikbkap - remain unknown. To better understand the normal function of IKAP and in an effort to generate a mouse model for FD, we have targeted the mouse Ikbkap gene by homologous recombination. We created two distinct alleles that result in either loss of Ikbkap expression, or expression of an mRNA lacking only exon 20. Homozygosity for either mutation leads to developmental delay, cardiovascular and brain malformations, accompanied with early embryonic lethality. Our analyses indicate that IKAP is essential for expression of specific genes involved in cardiac morphogenesis, and that cardiac failure is the likely cause of abnormal vascular development and embryonic lethality. Our results also indicate that deletion of exon 20 abolishes gene function. This implies that the truncated IKAP protein expressed in FD patients does not retain any significant biological function.

## Introduction

Familial Dysautonomia (FD), also known as "Riley-Day syndrome" or "hereditary sensory and autonomic neuropathy type III" (MIM 223900), is an autosomal recessive disorder that affects 1/3,600 live births in the Ashkenazi Jewish population. At birth, babies with FD display poor muscle tone, weak or absent suck, respiratory congestion due to misdirected swallow, and difficulty in maintaining body temperature. Pathological findings from adults and children indicate that within the peripheral nervous system, individuals with FD suffer from abnormal or incomplete neuronal development as well as progressive neuronal degeneration [1]-[3]. The criteria for diagnosis of FD in older children are: absence of fungiform papillae on the tongue, absence of flare after intradermal injection of histamine, decreased or absence of deep-tendon reflexes, absence of overflow emotional tears as well as impaired pain and temperature perception [4]–[9]. Other clinical features include gastrointestinal dysfunction, gastroesophageal reflux, vomiting crises, recurrent pneumonia, scoliosis and postural hypotension. In addition to pulmonary complications, patients sometimes die suddenly apparently due to cardiac asystole. Despite advances in patient care, progressive autonomic dysfunction eventually leads to premature death, with only 50% of patients reaching the age 40 [3].

Mutations in the Ikbkap gene (mapped to human chromosome 9q31) were shown to be the cause of FD [10]–[11]. In humans, the Ikbkap gene covers a 68 kb genomic sequence containing 37 exons and encodes a 150 kD protein named IKAP. In FD, the major haplotype (representing >98% of the FD cases) is associated with a T to C transition in position 6 of the donor splice site of intron 20 of the Ikbkap gene. This mutation results in the generation of an mRNA in which exon 20 (74 bp) is spliced out, along with intronic sequences, causing a frameshift and producing a truncated protein of 79 kD [10], [11]. This aberrant splicing however is not fully penetrant since wild-type (WT) Ikbkap mRNA is also observed in all tissues, albeit at severely reduced levels [11], [12].

IKAP (Inhibitor of kappaB kinase complex associated protein) was initially identified and named on the basis of its reported ability to bind IκB kinases and to assemble these proteins into an active kinase complex [13]. However, a later study failed to confirm the role of IKAP in cytokine-induced NF-κB activation, and instead suggested that IKAP may be directly involved in RNA polymerase II transcription elongation [14]. In favor of this hypothesis, IKAP or IKAP/Elp1 (due to the high homology with the yeast elongator ELP1), co-purifies with the human Elongator complex [15], [16], and was shown to bind the coding sequences of genes that are down-regulated in FD fibroblasts [17]. On the other hand, IKAP is mainly a cytoplasmic protein [13], [17], suggesting that, in addition to its putative role in transcriptional elongation, it may also be involved in other cellular processes. In support of this notion, cytosolic IKAP co-purifies with proteins involved in cell migration and cytoskeleton organization [19], and IKAP also associates with JNK and enhances JNK-mediated stress signaling [18]. In addition, other putative roles for IKAP as a cytoplasmic protein include exocytosis and tRNA modification as suggested based on the functions of its yeast homolog (ELP1) [20]–[22].

The above-mentioned studies suggest that IKAP may have distinct cellular functions, but the mechanism(s) altered resulting in FD remain to be elucidated. So far, there are no animal models that recapitulate the molecular and pathological findings of FD, thus a mouse model for FD would be an invaluable tool to dissect the functions of IKAP and understand the pathological findings of FD. In the mouse, the Ikbkap gene is located on chromosome 4 in a region that is syntenic to human chromosome 9q31 [23], [24]. The mouse IKAP protein shows high degree of homology with the human IKAP, with 80% aminoacid identity. The consensus donor splice site of intron 20, which is mutated in the major FD haplotype, is also conserved in the mouse [24]. These findings suggest that IKAP is likely to play a similar role in the mouse as in humans.

As part of an ongoing effort to generate a mouse model for FD, and to further understand the role(s) of IKAP in embryonic and postnatal development, we have introduced two types of mutations in the Ikbkap gene, resulting in either loss of expression of Ikbkap, or expression of an Ikbkap mRNA missing only exon 20. Homozygous mutants carrying either of the two mutations die around E10.5 and display identical phenotypic features, including developmental delay and abnormal brain and cardiovascular development. Our analyses indicate that IKAP is an essential protein for mouse embryonic development, that the truncated IKAP protein does not retain any biological function in embryogenesis, and that disruption of the canonical Smad4-dependent TGFβ signaling may underlie some of the developmental defects of mutant embryos.

## Results

### Targeted disruption of the Ikbkap allele

A mouse genomic DNA library (129Sv, Stratagene) was screened for clones containing the Ikbkap mouse sequence corresponding to human exons 18–21, and three clones were isolated and characterized.

**Figure 1:**
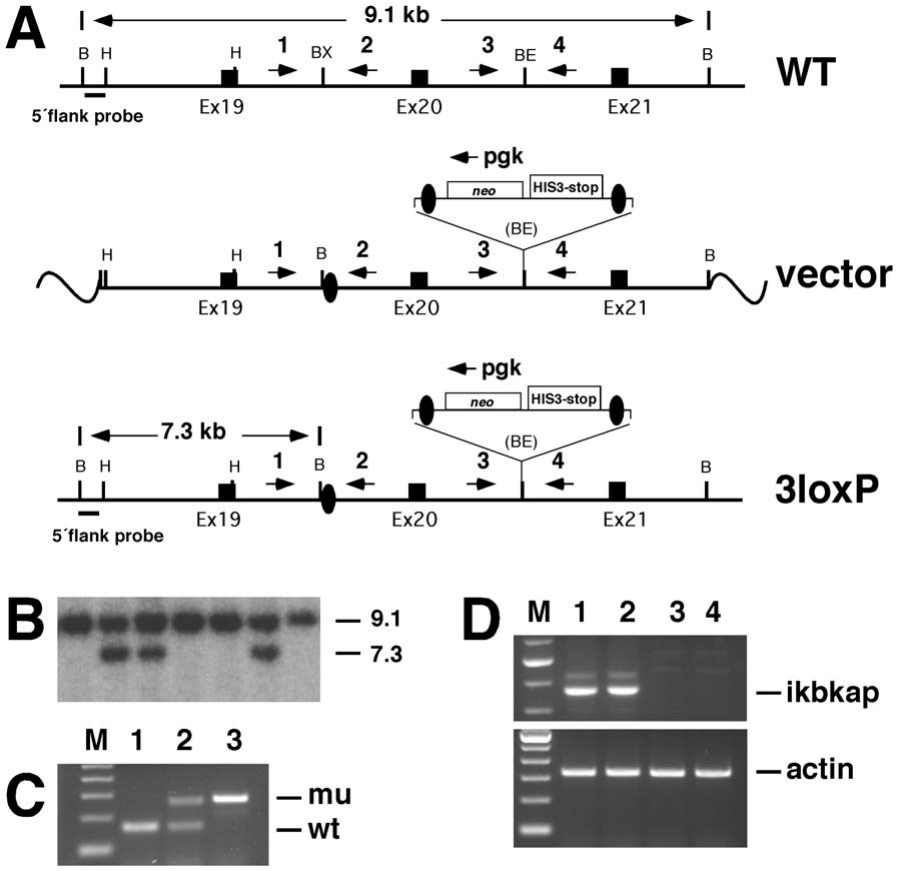
Targeted disruption of the mouse Ikbkap gene. (**A**) Schematic representation of wild-type allele (WT), Ikbkap targeting vector (vector), and Ikbkap targeted allele (3loxP). Exons are represented by black rectangles, and black ovals represent the loxP sites. The position of the 5′ external probe along with the sizes of the diagnostic fragments are indicated. The positions of the primers used for genotyping are indicated by numbered arrows (1, 2, 3, 4), also the position of the pgk oligo is marked by an arrow above the neo cassette. A loxP flanked neostop cassette (neo HIS3-stop) is also indicated. Restriction sites shown on the schematic are: Bam HI (B), HindIII (H), BstXI (BX) and BstEII (BE). (**B**) Southern blot of WT (lanes 1,4, 5, and 7) and targeted (lanes 2,3, and 6) ES clones, DNA digested with BamHI and probed with the 5′ flank probe. (**C**) Genotyping of progeny derived from Ikbkap^3loxP/+^ intercross. Genomic DNA from WT (lane 1), heterozygous (lane 2) and homozygous (lane 3) progeny was amplified by PCR using the primers Ikap1for and Ikap2rev 2. (**D**) RT-PCR on reverse transcribed RNA from WT (lanes 1 and 2) and Ikbkap^3loxP/3loxP^ (lanes 3 and 4) embryos using the primers Ikap18for and Ikap23rev is depicted (top panel). β-actin amplification (bottom panel) was performed in parallel to verify that equal amounts of starting material were used in each reaction.

**Figure 2:**
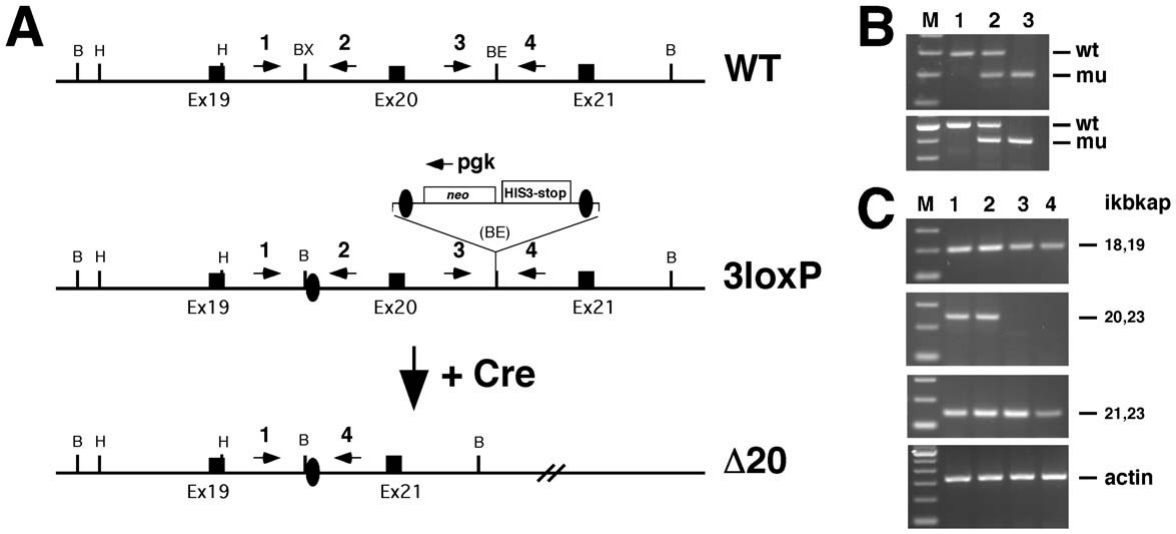
Generation of an Ikbkap allele lacking exon 20. (**A**) Schematic representation of wild-type allele (WT), Ikbkap^3loxP^ allele (3loxP), and Ikbkap allele lacking exon 20 after Cre-mediated recombination (Δ20). Exons are represented by black rectangles, and black ovals represent the loxP sites. The positions of the primers used for genotyping are indicated by numbered arrows (1, 2, 3, 4), also the position of the pgk oligo is marked by an arrow above the neo cassette. A loxP flanked neostop cassette (neo HIS3-stop) is also indicated. Restriction sites shown on the schematic are: Bam HI (B), HindIII (H), BstXI (BX) and BstEII (BE). (**B**) Genotyping of progeny derived from Ikbkap^Δ20/+^ intercross. Genomic DNA from WT (lane 1), heterozygous (lane 2) and homozygous (lane 3) progeny amplified by PCR using the primers Ikap1for Ikap4rev (top panel) and Ikap19for and Ikap4rev (bottom panel). (**C**) Expression of Ikbkap was assessed by RT-PCR in WT (lanes 1 and 2) and homozygous (lanes 3 and 4) embryos using the primers Ikap18for and Ikap19rev (top panel), Ikap20for and Ikap23rev, Ikap21for and Ikap23rev (middle panels). β-actin amplification (bottom panel) was performed in parallel to verify that equal amounts of starting material were used in each reaction.

The targeting construct used to disrupt the mouse Ikbkap gene was designed in such a way to include a loxP-flanked neostop cassette [25] in intron 20 and a loxP site in intron 19 (see materials and methods and Figure 1A). This strategy was used since it represents the most efficient way of generating three mutant alleles with one targeting vector [25]. In summary this strategy is advantageous since: a) by itself the insertion of the neostop cassette in the gene destabilizes the mRNA resulting in severe reduction or elimination of gene expression, as has been seen with other genes [25]–[27], b) Cre-mediated recombination between the external pair of loxP sites would generate a deletion of exon 20 (see below and Figure 2A), and c) if recombination deletes only the neostop cassette, a conditional inactivation allele is generated in which deletion of exon 20 can then be controlled in a spatio-temporal manner.

After electroporation into ES cells and selection with G418, correctly targeted clones were identified by Southern blot analyses (Fig. 1B). Targeted clones carrying the neostop cassette and the three loxP sites (*Ikbkap^3loxP/+^*) were injected into E3.5 C57BL/6 blastocysts. Chimeric mice generated from the injections transmitted the targeted allele to their progeny. Mice heterozygous for the *Ikbkap^3loxP/+^* allele were normal and fertile.

### Loss of Ikbkap gene function results in early embryonic lethality

Heterozygous *Ikbkap^3loxP/+^* mice were intercrossed to generate homozygous *Ikbkap^3loxP/3loxP^* mice. Genotyping of the resulting progeny at P10 and P0 revealed that no homozygous live-born pups could be recovered from these matings, indicating that the mutation was incompatible with normal embryonic development. To determine the timing and cause of the embryonic lethality, pregnant females were dissected at different embryonic stages. At E9.5-E10.5, *Ikbkap^3loxP/3loxP^* embryos were recovered at Mendelian ratio, but no live mutant embryos were obtained after E11.5.

To confirm that the *Ikbkap^3loxP/3loxP^* is a null allele, embryos derived from *Ikbkap^3loxP/+^* intercrosses were dissected at E9.5 and E10.5. After genotyping of extraembryonic tissues, total RNA from WT and *Ikbkap^3loxP/3loxP^* mutants was reverse-transcribed and subjected to PCR amplification for mouse Ikbkap using pairs of primers that span exons 18–23. Our results show that there is no Ikbkap expression in *Ikbkap^3loxP/3loxP^* mutants, due to the destabilization of the mRNA caused by the neostop cassette in intron 20 (Fig. 1D).

### Deletion of exon 20 results in a non-functional allele

In FD patients, the underlying mutation (responsible for over 98% of FD cases) causes skipping of exon 20, leading to a frame shift and the expression of a truncated protein of 79 kD. Although the truncated protein is detected in tissues where skipping of exon 20 is predominant [10], it is not known whether this truncated protein retains any of the normal IKAP function or if it acquires new properties. To address this question we generated mice carrying a deletion of exon 20 in the Ikbkap gene (*Ikbkap^Δ20/+^*; see Figure 2A). For this purpose *Ikbkap^3loxP^* ^/+^ mice were crossed with a Cre “deletor” line [28]. Progeny carrying both the targeted Ikbkap allele and the Cre transgene underwent successful deletion of both the exon 20 and the neo cassette, and transmitted the *Ikbkap^Δ20/+^* allele to their progeny (Fig. 2B). As expected, *Ikbkap^Δ20/+^* mice were viable and fertile. However, intercross of *Ikbkap^Δ20/+^* mice did not yield viable progeny (no homozygous mutants was found at P1, data not shown). Dissections of pregnant female mice at different time points revealed that *Ikbkap^Δ20/Δ20^* embryos died around E11.5 and were morphologically indistinguishable from *Ikbkap^3loxP/3loxP^* embryos (see below). Ikbkap expression in *Ikbkap^Δ20/Δ20^* and control embryos was assessed by RT-PCR on total RNA at E10.5 using sets of primers that amplify through exons 18 to 23. As shown in Figure 2C, Ikbkap mRNA could be amplified from *Ikbkap^Δ20/Δ20^* embryos when primers spanning exons 18/19 or 21/23 were used, but no product was amplified when one of the primers was located in exon 20, confirming the production of a stable mRNA lacking only exon 20.

### Ikbkap mutant embryos are developmentally delayed and display cardiovascular and brain malformations

To determine the nature of the developmental abnormalities of *Ikbkap^3loxP/3loxP^* and *Ikbkap^Δ20/Δ20^* mutants, and the cause of the embryonic lethality, embryos derived from the respective heterozygous intercrosses were dissected at different stages of gestation from E8.5 to E10.5. Both types of mutations produced embryos with practically identical phenotypes at all stages examined (see below, table 1 and data not shown), confirming that the Ikbkap^Δ20^ allele does not retain any Ikbkap biological activity.

**Table 1:** – *Ikbkap^Δ20/Δ20^* mutant embryos are identical to *Ikbkap^3loxP/3loxP^*embryos.

	E9.5			E10.5		
Matings	WT	heterozygous	Mutant a,b	WT	heterozygous	Mutant a,c
*Ikbkap^3loxP/+^*X *Ikbkap^3loxP/+^*	8	20	7	12	23	11(2)
*Ikbkap^Δ20/+^* X *Ikbkap^Δ20/+^*	10	21(1)	8(1)	9	24	10 (3)
*Ikbkap^3loxP/+^*X *Ikbkap^Δ20/+^*	4	7	3	7	12	5 (1)

Total number of embryos recovered at each stage is indicated; number of dead embryos is included in parenthesis.

a Mutant homozygous (*Ikbkap^3loxP/3loxP^* or *Ikbkap^Δ20/Δ20^*) or compound heterozygous (*Ikbkap^3loxP/Δ20^*) embryos were recovered at Mendelian ratio at E9.5 and E10.5.

b The majority of the embryos recovered were alive and appeared to be one day delayed.

c ∼75% of the embryos were alive, appeared to be one day delayed and exhibited impaired cephalic development and severe cardiac abnormalities, see results.

**Figure 3:**
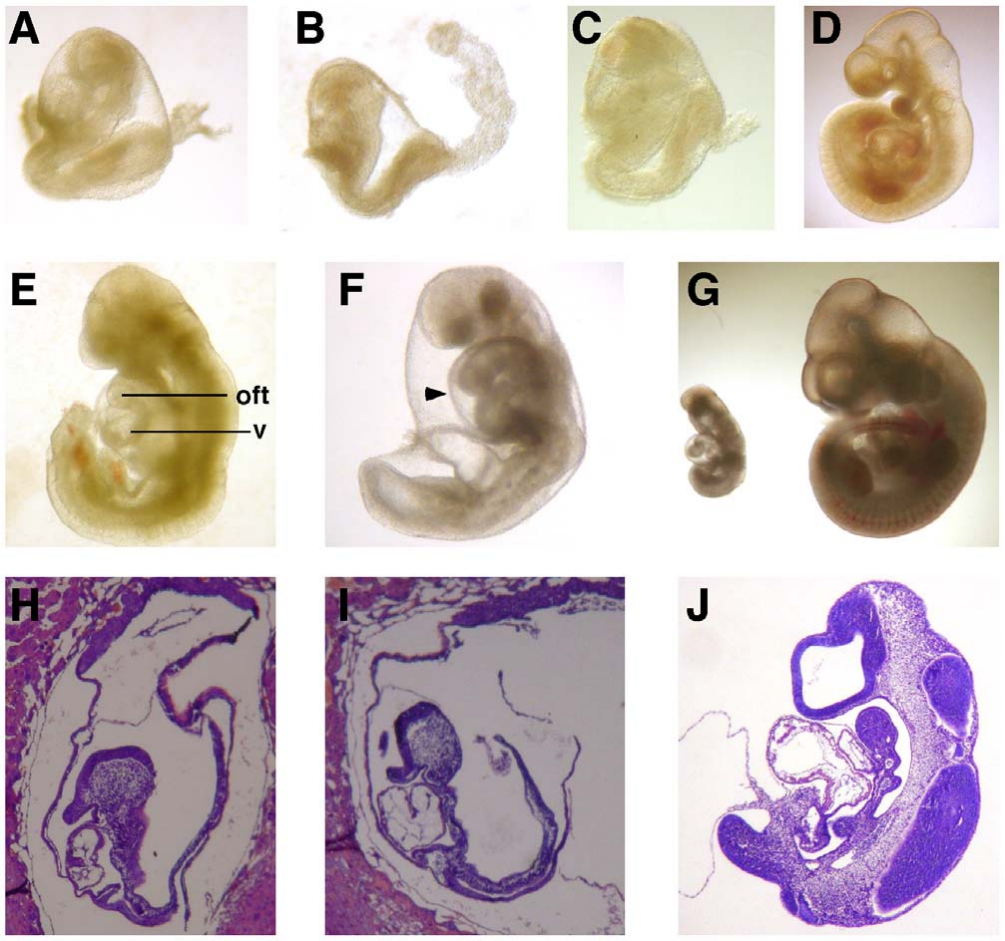
Phenotypical features of mutant Ikbkap and control embryos. (**A**) Wild-type embryo at E8.5. Mutant Ikbkap^3loxP/3loxP^ (**B**) and Ikbkap^Δ20/Δ20^ (**C**) embryos at E9.5, photographed in the same magnification as the embryo shown in A. Note that mutant embryos at this stage closely resemble the WT E8.5 embryo in size and external morphology. (**D**) WT embryo at E9.5 (different magnification than embryos in A–C). Note that looping of the heart has already occurred at this stage. Ikbkap^3loxP/3loxP^ (**E**) and Ikbkap^Δ20/Δ20^ (**F**) embryos at E10.5. In the mutants, anterior cephalic development is severely compromised. In Ikbkap mutant embryos the forward growth of heart ventricle (v) occurred in the absence of looping morphogenesis. Note the misaligned position of the outflow tract (oft) in mutant embryos compared to the embryo in D and the enlarged pericardial sac (arrowhead). (**G**) E10.5 Ikbkap^Δ20/Δ20^ (left) and WT (right) littermates photographed together. (**H–J**) H&E-stained sagital sections of E8.5 WT (**H**), E9.5 Ikbkap^3loxP/3loxP^ (**I**) and E9.5 wild-type (**J**) embryos. Note that at E9.5 cephalic and heart development of mutant embryos is similar to the WT E8.5 embryo in H.

Morphological and histological analyses at E8.5 revealed that mutant embryos were already developmentally delayed (data not shown). At E9.5 Ikbkap mutant embryos were significantly smaller than their WT littermates and resembled WT E8.5 embryos in size and general morphology (Fig. 3A–C), suggesting that development of mutant embryos was approximately one day delayed relative to their control littermates (Fig. 3D). Histological analyses of mutant embryos at this stage confirmed that in mutant embryos cephalic and cardiac development was similar to that of E8.5 control embryos (Fig. 3H–J).

By E10.5, development of the mutant embryos had progressed, and they had reached several of the developmental milestones of E9.5 WT embryos: for instance, neural fold closing and turning of the embryo had occurred, the lower mandibular process of the first branchial arch had developed, the otic pit had become clearly visible, and development of the limb buds had begun. Marked differences however were noticed at this stage: compared to WT E9.5 embryos, E10.5 mutant embryos were pale and their yolk-sacs poorly vascularized, they did not develop a second branchial arch, and exhibited severe brain and cardiac developmental abnormalities (Fig. 3D–G and data not shown). Gross morphological inspection of the embryos indicated that although midbrain and hindbrain regions appeared similar to that of E9.5 WT embryos, development of the forebrain and in particular of the telencephalon was markedly compromised.

The heart appeared severely abnormal in mutant embryos, and in embryos recovered dead at E10.5 heart development had not progressed further than an E8.5-like primitive heart (data not shown). Hearts of mutant embryos that were alive at E10.5 (heart beating) exhibited abnormal looping and dilated pericardium sacs (Fig. 3E–G), although their development had progressed and constriction of the atrial ventricular canal could be observed. At E11.5, all mutants displayed severe pericardial effusion accompanied by loss of heart-beat (data not shown). These observations indicate that heart failure is most likely the cause of death of Ikbkap mutant embryos.

### Histological analysis of Ikbkap mutants

**Figure 4:**
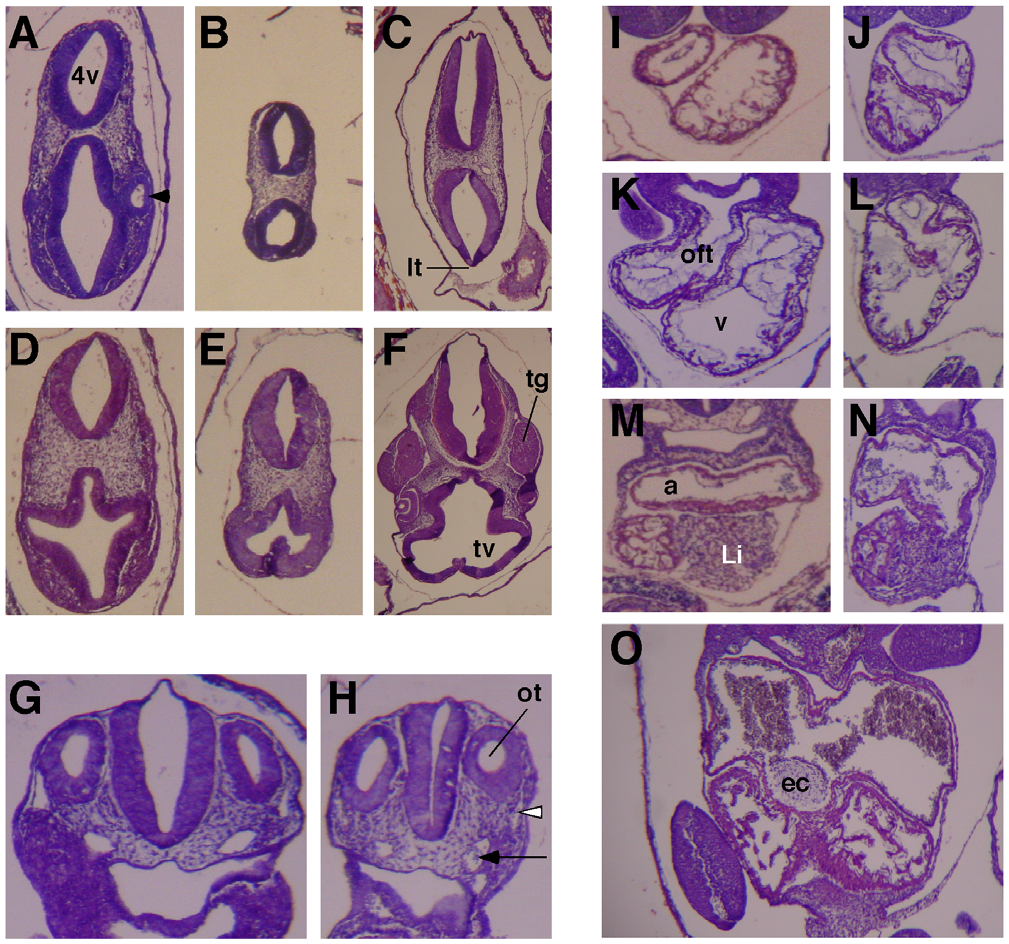
Histological analyses of mutant Ikbkap and control embryos. (**A–F**) H&E-stained transverse sections through the head of E9.5 WT (**A** and **D**), E10.5 Ikbkap^3loxP/3loxP^ (**B** and **E**) and E10.5 WT (**C** and **F**). A, B, D and E are shown in the same magnification for comparison. Note that the telencephalic vesicle (tv) of the mutant embryo (E) is poorly developed compared to D and F. Note also that the lamina terminalis (lt) is absent in the mutant (**B**), and that there is no evidence of formation of the optic vesicle (arrowhead). In contrast, the 4^th^ ventricle (4v) is well formed and comparable to the control embryo in A. tg = trigeminal ganglion. (**G** and **H**) H&E-stained transverse sections at the level of the otic vesicle of E9.5 WT (**G**) and E10.5 Ikbkap^3loxP/3loxP^ (**H**) embryos. Note that in the E10.5 mutant the otic vesicle (ot) has separated from the overlying surface ectoderm, a landmark of normal development of E9.5 stage (G). Note that although the dorsal aorta (arrow) and cardinal vein (white arrowhead) are visible in the mutant in H they are hypoplastic compared to the embryo in G. (**I–N**) H&E-stained transverse sections through the heart (rostral to caudal) of E9.5 WT (**I**, **K** and **M**) and E10.5 Ikbkap^3loxP/3loxP^ embryos (**J**, **L** and **N**). Note the abnormal relative position of the outflow tract (oft) in the mutant heart (**L**) compared to a normal embryo (**K**). The myocardium and also the level of trabeculation of the ventricle in the mutant (**J** and **L**) appear comparable to an E9.5 control embryo (**I** and **K**). v = ventricle, a = atrium, Li = liver. Note that development of a WT E10.5 heart has proceeded significantly (**O**) with extensive trabeculation, separation of left and right ventricles, and formation of the endocardial cushion (ec).

To further analyze the brain and cardiovascular developmental defects of mutant embryos, we performed histological analyses on serial sections of E9.5 and E10.5 control and mutant *Ikbkap^3loxP/3loxP^* and *Ikbkap^Δ20/Δ20^* embryos. Histological examinations confirmed that anterior development was particularly affected in *Ikbkap^3loxP/3loxP^* and *Ikbkap^Δ20/Δ20^* mutant embryos at E10.5. While in E9.5 WT embryos the telencephalic vesicle was expanded and the lamina terminalis (the most rostral part of the telencephalon) was evident, in mutant embryos at E10.5 the telencephalic vesicle was poorly developed and the lamina terminalis could not be identified (Fig. 4A–F, and data not shown). Formation of other anterior structures also appeared compromised as there was no evidence of formation of the optic vesicle in mutant embryos at E10.5, although this structure is readily visible in E9.5 WT embryos (Fig. 4A, B, and D, E). Histological analyses of diencephalon, midbrain and hindbrain regions of mutant E10.5 embryos indicated that these structures were grossly similar to the corresponding regions of WT E9.25-E9.5 embryos although less expanded (Fig. 4, and data not shown). Together these observations suggest that although IKAP is not required for proper formation of the neuroepithelium itself, or for neural tube closure, it is required for development of the forebrain and other anterior structures.

Cardiac and vascular development was also severely compromised in *Ikbkap^3loxP/3loxP^* and *Ikbkap^Δ20/Δ20^* mutant embryos. Although the dorsal aorta and cardinal veins could be easily identified, they were invariably hypoplastic (Fig. 4G and H). In agreement with a probable developmental delay of 1 day of gestation, mutant hearts at E10.5 had not formed the endocardial cushion, nor displayed the level of ventricular trabeculation observed in WT E10.5 embryo hearts, but rather ventricular development appeared comparable to the hearts of WT E9.5 embryos (Fig. 4I-O). However, consistent with the morphological observation of abnormal heart looping, serial transverse sections through the heart of mutant and control embryos at E10.5 and E9.5 respectively, confirmed that the outflow tract was misaligned in the mutant embryos (Figure 4I–L). These results indicate that IKAP is essential for cardiovascular development during early embryogenesis.

### Molecular analyses of Ikbkap mutants

**Figure 5:**
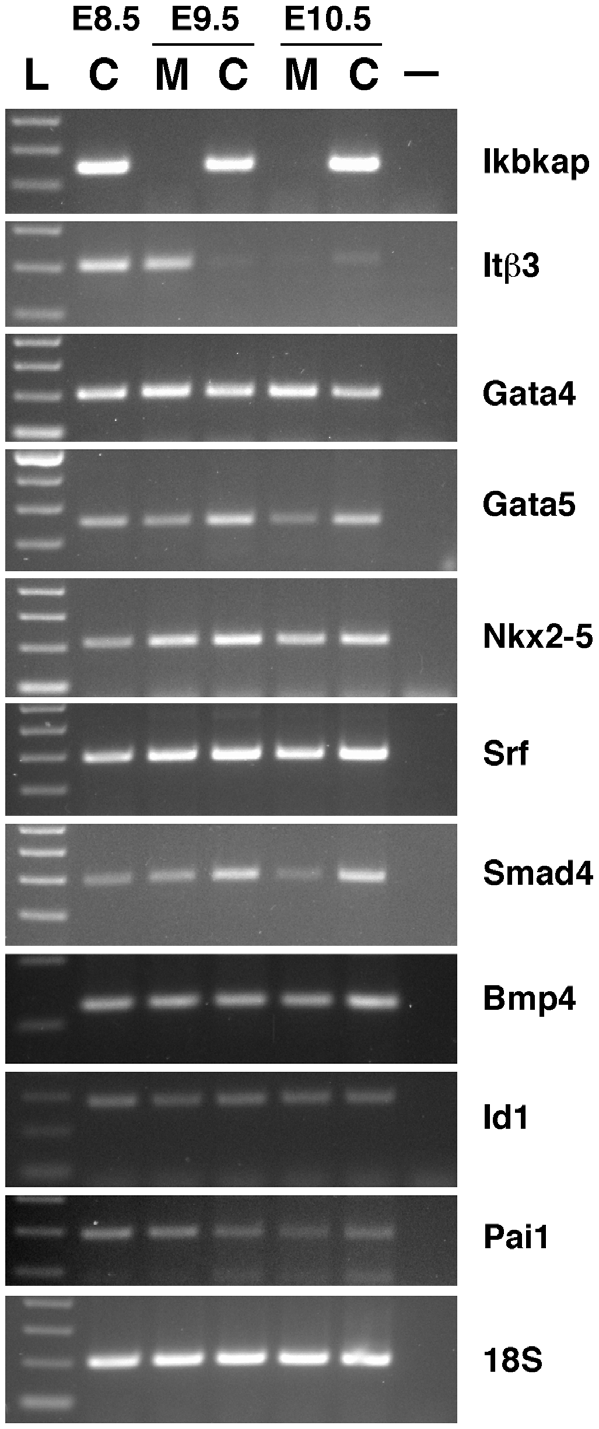
Semi-quantitative RT-PCR analyses. Equal amount of total RNA from control (C) and mutant (M, Ikbkap^Δ20/Δ20^) embryos at the indicated stages (E8.5-E10.5) was used for cDNA synthesis in the presence of reverse transcriptase (RT) and the resulting cDNA was used for amplification of the specified genes (see details in Materials and Methods). Similar results were obtained for Ikbkap^3loxP/3loxP^ embryos (data not shown). For negative control (-), reverse transcription reaction was performed in the absence of RT using equal starting amounts of total RNA as in all other reactions. Amplification of the rRNA 18S (bottom panel) was used as internal control for normalization of cDNA template quantity in the PCR reactions. As marker 100 bp ladder (L) is also shown. Lack of Ikbkap amplification with oligos Ikbkap20for and Ikbkap23rev in the upper most panel reconfirms the genotyping results for the mutant (M) Ikbkap^Δ20/Δ20^ embryos used for the depicted analyses.

To investigate the molecular pathways underlying the abnormal heart morphogenesis of the mutant embryos, we analyzed expression of multiple genes involved in heart development. We first examined expression of the integrin β3 gene (Itβ3) whose expression is restricted to the primitive heart at E8.5 and is downregulated at E9.5-E10.5, as heart development progresses [29], [30]. Consistent with the histological analyses, Itβ3 expression was observed in E8.5 WT and E9.5 Ikbkap mutant embryos, and was downregulated at later stages in both WT and mutants (Fig. 5), confirming that at E9.5 mutant embryos display a primitive heart similar to that of a WT E8.5 embryo. Downregulation of Itβ3 transcripts at E10.5 in mutant embryos is also consistent with the progression of heart development in the mutants.

Heart morphogenesis and development depends on a complex regulatory network, and phenotypic analyses of mutant mice with abnormal heart development has uncovered the involvement of several genes in this process such as members of the Nkx2-5 homeobox transcription factor network (Nkx2-5, Gata4, Gata5, Gata6, and Srf), and of the Bmp/Tgfβ signaling pathway, in which Smad4 acts as a key transcriptional co-activator [31]–[41]. Although semi-quantitative RT-PCR analyses on E8.5 - E10.5 control and mutant embryos did not reveal significant differences in expression levels of Gata4, Nkx2-5 and Srf between mutant and control embryos, expression of Smad4 and Gata5 was significantly reduced in E10.5 mutants (about five-fold and four-fold reduction respectively) compared to either E9.5 or E10.5 controls (Fig. 5). The severe reduction in Smad4 expression prompted us to further investigate the impact of IKAP loss on TGFβ signaling. For this purpose, we analyzed the expression of Bmp4 (an essential TGFβ superfamily member), and of two downstream direct targets of Smad4, Inhibitor of DNA binding 1 (Id1) and plasminogen activator inhibitor type 1 (Pai1), which are downregulated in embryos lacking Smad4 in endothelial cells [38]. Although Bmp4 and Id-1 mRNA levels were unaltered in mutant embryos, Pai-1 expression was considerably reduced at E10.5 in mutant embryos compared to either E9.5 or E10.5 WT embryos (Figure 5). Taken together, our results indicate that loss of IKAP partially impairs Smad4-dependent TGFβ signaling in embryogenesis.

## Discussion

Although it is generally believed that severe reduction of IKAP expression is the leading or sole cause of FD [3], [12], [42], [43], to date there is no information regarding the possible biological role(s) of the truncated protein, which is produced due to skipping of exon 20 [10]. In order to understand the essential roles of IKAP during embryonic development and to investigate the biological consequences of exon 20 deletion, we have generated two mutations in the mouse Ikbkap gene, and created two distinct alleles that result in either loss of Ikbkap expression, or expression of an mRNA lacking only exon 20. Our results show that in the mouse loss of Ikbkap expression or expression of a truncated IKAP protein due to deletion of exon 20 have identical biological consequences – developmental delay and early embryonic lethality due to abnormal cardiovascular development. These results prove for the first time that the truncated IKAP protein does not retain any significant biological function, at least during embryogenesis.

In agreement with our findings, Chen and collaborators [44] - using a gene-trap ES cell line obtained from Bay Genomics/IGTC - have recently demonstrated that inactivation of Ikbkap expression in the mouse leads to early embryonic lethality accompanied by severe growth retardation and developmental and vascular abnormalities. However, their analyses relied solely on morphology, and from their description and photographic documentation it is difficult to ascertain the range and degree of developmental abnormalities of the mutant embryos. In addition, the molecular analyses presented was restricted to one stage only (E8.5) and did not take into account the already existing developmental delay of Ikbkap knockout embryos, making the interpretation of the differences in transcriptional profiles problematical.

Here we provide morphological, histological and molecular evidence that show that loss of a functional IKAP protein leads to a one day developmental delay, and that although mutant embryos reach several milestones of a typical E9.5 embryo, they display anterior cephalic developmental defects, and cardiovascular abnormalities with cardiac failure being most likely the leading cause of early embryonic lethality.

Delayed development is an early feature of mutant embryos already observed at E8.5 (data not shown), and by E9.5, although development has progressed, mutant embryos are similar to E8.5 WT embryos as seen by morphological and histological analyses (Fig. 3) as well as by RT-PCR amplification of the stage-specific cardiac marker Itβ3 (Fig. 5). By E10.5 however, although mutant embryos had reached some of the milestones of E9.5 control embryos, considerable abnormalities could be easily observed, with cephalic and cardiac development being significantly compromised (Fig. 3 and 4).

Although the cause of the developmental delay of IKAP mutant embryos requires further investigation, it is possible that IKAP is required for regulation of apoptotic programmed cell death and/or cell proliferation during early embryogenesis. Embryos lacking the zinc finger protein ZFR [45] display embryonic developmental delay (as our Ikbkap mutant embryos), a delay that is caused by increased cell death with concomitant decreased cell proliferation in the epiblast at E6.0–E6.5. Increased cell death at the same developmental stage leading to developmental delay was also reported in embryos lacking the human Bloom syndrome gene [46]. Also, further support for a putative role of IKAP in regulation of cell death or proliferation is enhanced by the findings that elimination of IKAP *in vitro* enhances basal expression of pro-apoptotic p53-dependent genes [47] and decreases expression of genes involved in cell proliferation [17].

Anterior development is also compromised in the absence of IKAP (see Figures 3 and 4). While the midbrain and hindbrain regions of E10.5 mutant embryos were similar to E9.5 WT embryos, development of the forebrain - and more specifically of the telencephalon - was severely compromised (Fig. 4). Although by E9.5 development of the neuroepithelium in mutant embryos was comparable to WT E8.5 embryos and did not exhibit signs of increased cell death by H&E staining (Fig. 3, and data not shown), decreased cell proliferation or aberrant patterning signals could account for impaired anterior development. Of importance is our finding that Smad4 expression is severely reduced in IKAP mutant embryos. Smad4, a key mediator of Bmp/Tgfβ signaling [35], [36], is directly implicated not only in cardiovascular development (see below) but also in head and brain development, as embryos lacking Smad4 expression in the embryo proper exhibit anterior truncations [35]. It is possible therefore that the abnormal formation of the telencephalon and of other anterior structures in IKAP mutants is due to downregulation of Smad4-dependent signaling (see Figure 5).

Even though the developmental defects of the mutant embryos could be attributed to the need for IKAP in multiple processes, cardiac failure is the most likely explanation for their early death and could also explain their poor vascularization. Secondary effects of hypoxia due to cardiac failure could also be responsible for the abnormal development of anterior structures and including the second pharyngeal arch. In vertebrate embryos, the cardiovascular system is the first organ system to develop, and in the mouse, the hemodynamic force and blood flow generated by embryonic heart-beat - between E8.5 and E9.5 - induce remodeling of primitive vessels of the yolk sac to form a branched network of large and small-caliber vessels, and also vascular remodeling and angiogenesis in the embryo proper [48]–[53]. While a functional primitive heart and large vessels are formed properly (albeit with one day delay) in the absence of IKAP (Fig. 4), the large vessels were invariably hypoplastic and vascular remodeling of the yolk sac was severely compromised. Although many genes that are required for vascular remodeling have a cell-autonomous role in endothelial cells, mutations that affect primarily cardiac development also cause abnormal vascular remodeling [51]. In IKAP mutant embryos, heart looping - which occurs between E8.75 and E9.25 in WT embryos [54], [55] - is abnormal, and despite the fact that growth of the ventricle occurs in the absence of looping morphogenesis, there is absence of atrioventricular canal and misalignment of the outflow tract (Fig. 3 and 4). Similar cardiac abnormalities were shown to be the primary cause of early embryonic lethality and vascular remodeling defects in embryos lacking cardiac expression of Nkx2-5 [31], [32], Srf [41], and Tgfrb2 [56]. Notably, impaired contractility by itself was shown to be sufficient to abolish vascular remodeling of the yolk sac in mice [53]. These observations however do not rule out the possibility that IKAP may also have a cell-autonomous function in endothelial cells and as a result IKAP may also play a direct role in vascular remodeling.

Since cardiovascular defects are prominent in Ikbkap null embryos, we evaluated the impact of loss of IKAP in expression of several genes involved in cardiac morphogenesis and vascular remodeling. Surprisingly, we found that expression of several major genes involved in cardiac development, including Gata4, Nkx2-5 and Srf, was normal in E9.5 and E10.5 mutant embryos. Interestingly, and while expression of Gata5 was significantly lower in E9.5 mutants, expression of Gata5 and Smad4 was severely reduced by E10.5 (Fig. 5). The essential roles of Gata5 during cardiogenesis in mice have only recently started to emerge. Although it has long been reported that mice lacking Gata5 are viable and do not display cardiac defects [57], Singh and collaborators have recently shown that Gata4^+/-^; Gata5^-/-^ mutants die at mid-gestation with profound cardiovascular abnormalities [58], suggesting a cooperative interaction between these transcription factors during heart development. However, since expression levels of Gata4 were unaltered in Ikbkap mutants (Fig. 5), the biological significance of Gata5 downregulation in IKAP embryos remains to be elucidated. In contrast, the essential roles of Smad4-dependent TGFβ signaling in cardiovascular development has been extensively documented. For instance, loss of Smad4 expression in neural crest cells, cardiomyocytes, or endothelial cells result in severe cardiovascular defects and embryonic lethality [38], [39], [59]. In addition, disruption of TGFβ signaling during cardiogenesis leads to aberrant heart looping and impaired remodeling of the atrioventricular canal [56]. Our results indicate that reduction of Smad4 expression in mutant embryos leads to significant downregulation of its downstream target Pai1, suggesting that downregulation of Smad4 expression and consequent disruption of TGFβ signaling may be the primary cause of the cardiovascular defects of IKAP mutant embryos.

Our findings also shed light to potential mechanisms underlying FD pathology. First, we have shown that the truncated IKAP protein expressed in FD due to exon 20 skipping does not retain significant biological function, at least during embryogenesis. This implies that most likely the reduction of IKAP expression is the only cause of sensory and autonomic developmental defects of FD patients, and also that even a very low level of full-length IKAP (as seen in FD patients) is sufficient to carry on embryonic development up to birth. Second, apart from its essential roles in anterior and cardiovascular development, Smad4-dependent TGFβ signaling has also been shown to play an essential role in development and maintenance of sensory and autonomic systems. It has been shown that conditional inactivation of Smad4 in neural crest cells does not affect neurogenesis and ganglia formation but results in reduction of proliferation and noradrenergic differentiation in sympathetic ganglia, and elimination of TrkA-positive subpopulations in trigeminal ganglia [60], [61]. Hence, in view of the requirement of IKAP for regulation of Smad4 expression, it is possible that disruption of TGFβ signaling in neural crest cells may contribute to FD pathology. Unfortunately, the developmental delay associated with possible secondary effects of hypoxia, and early death of mutant embryos lacking IKAP precludes the analyses of the developmental status of the peripheral nervous system at this point. Conditional inactivation of IKAP in specific cell populations, and in particular in neural crest cells, should elucidate the role(s) of IKAP in PNS development.

## Materials and Methods

### Construction of the targeting vector

An RT-PCR product (∼660 bp corresponding to position 2360-3025 of the human IKAP cDNA, accession number AF153419) was used as a probe to screen a mouse genomic DNA library (Stratagene) for clones containing the Ikbkap mouse sequence corresponding to human exons 18-21. We identified and picked 3 phages that hybridized with our probe. Inserts were isolated, cloned in a cosmid vector and characterized. A clone with an 11 kb insert containing exon 20 was partially sequenced and characterized with restriction endonucleases. A 5.8 kb HindIII fragment and a 3.3 kb HindIII/NotI fragment of the Ikbkap genomic clone were initially subcloned separately into pBluescript and used as backbone for the generation of the targeting vector. A loxP flanked neostop cassette [25] was then cloned into the unique BstEII site of intron 20, and a loxP site was cloned into the BstXI site in intron 19.

### Generation of Ikbkap mutant mice

The targeting vector was linearized and introduced by electroporation into W9.5 ES cells grown on mitomycin C-treated G418-resistant primary mouse fibroblasts. After selection with G-418 resistant ES clones were picked and expanded. DNA purified from ES cells was analyzed by Southern blotting using a probe that distinguishes between the targeted (*Ikbkap^3loxP^*) and wild type alleles (Fig. 1A). A neo probe was then used to verify that single copy integration occurred in the targeted ES cells (data not shown).

Targeted ES cells were injected into the blastocoel cavity of E3.5 C57BL/6 embryos using standard procedures [62]. Germline chimeras were produced with three independent *Ikbkap^3loxP^* ^/+^ ES clones. Chimeras were backcrossed to C57BL/6 females that yielded heterozygous progeny for the Ikbkap mutant allele.

To generate mice in which only exon 20 is deleted (*Ikbkap^Δ20/+^*), *Ikbkap^3loxP^* ^/+^ mice were crossed with a Cre deletor line [28] maintained in C57BL/6 background. Progeny carrying both the targeted Ikbkap allele and the Cre transgene underwent successful deletion of exon 20 and the neo cassette, and transmitted the *Ikbkap^Δ20^* ^/+^ allele to their progeny (Fig. 2). All protocols for animal use were approved by the Institutional Animal Care and Use Committee of the University of Tennessee, and were in accordance with NIH guidelines.

### PCR assays for genotyping

For routine genotyping genomic DNA was prepared from tail biopsies or yolk sacs (embryos), and PCR amplification reactions were carried in the following conditions: 35 cycles consisting of 45 sec denaturation at 94°C, 45 sec annealing at 61°C, and 1 min extension at 72°C. The following primers were used for genotyping:

Ikap1for 5′-TGATTGACACAGACTCTGGCCA-3′, Ikap2rev 5′-GGAGATCCTCTAAGCAGCAGG-3′, Ikap3for 5′- CAGATTCGGAAGTGGCTGGAC-3′, Ikap4rev 5′-CTTTCACTCTGAAATTACAGGAA-3′, Ikap19for 5′-GAGAAATTCTGCGGAAAGTGGAA-3′ and pgk (pgk promoter sequence): 5′-GCCCGGCATTCTGCACGCTT-3′.

Diagnostic PCR reactions were the following:

Ikap1for and Ikap2rev amplify a 160 bp product from the wt allele, and a 290 bp product from the targeted *Ikbkap^3loxP^* allele; Ikap1for and Ikap4rev amplify a 390 bp product from the wt allele, and a 300 bp from the mutant *Ikbkap^Δ20^* allele; Ikap19for and Ikap4rev amplify a 605 bp from the wt allele and 500 bp for the *Ikbkap^Δ20^* allele.

### Histological analyses

For histology, decidua derived from heterozygous intercrosses were collected and fixed overnight in 4% (wt/vol) paraformaldehyde in phosphate-buffered saline (PBS); incubated for 24 hours at 4°C in PBS containing 0.25 M sucrose, 0.2 M glycine; dehydrated; cleared with toluene; and embedded in paraffin. Paraffin blocks were sectioned at 7 µm, mounted onto superfrost slides (Fisher), and selected slides were stained with hematoxylin and eosin. Staging of embryos and corresponding histological sections was performed according to Kaufmann [63].

### Genotyping of paraffin-embedded embryos

Genotyping of paraffin-embedded and sectioned material was carried out as described [64] with some modifications. Briefly maternal deciduas were scraped out and slides were deparaffinized in Citrisolv (Fisher), and re-hydrated in a series of decreasing alcohol concentrations. Deparaffinized sections were scraped and placed in eppendorf tubes containing 50 µl of 500 mM Tris pH 8.8, 10 mM NaCl, 20 mM EDTA, 3% Tween 20, supplemented with 1.5 mg proteinase K (PK)/ml. After overnight incubation in a 55°C water bath, samples were boiled for 10 min and 2 µl was used as template for PCR amplification under routine conditions for genotyping.

### RT-PCR assays

Embryos derived from *Ikbkap^3loxP/+^* and *Ikbkap^Δ20/+^* intercrosses were dissected at E8.5, E9.5 and E10.5. Yolk sacs were used for genotyping and embryos were snap frozen and stored at –80°C until needed. Total RNA was isolated from WT and mutant embryos using Trizol reagent (GIBCO-BRL) according to the manufacturer's instructions. For reverse transcription (RT) 1 µg total RNA was annealed with random hexamers, and first strand cDNA synthesis was carried out using Superscript III reverse transcriptase kit (Invitrogen). For assessment of gene expression, 1 µl of each RT reaction (equivalent to 50 ng of starting mRNA) was used for semi-quantitative PCR amplification. All PCR reactions were carried out in the same conditions: 45 sec denaturation at 94°C, 45 sec annealing at 61°C, and 1 min extension at 72°C.

The following pairs of primers were used: Ikap18for 5′-TCACGTCATTTGCTGTGTGTGAT-3′ and Ikap19rev 5′- CTTTGTGTCCTGGGGAACAACT-3′ (195 bp product); Ikap21for 5′ACCTCAATCTGATTCATGACCATA-3′ and Ikap23rev 5′-GGAGGGTACATGGTCTTTGTGA-3′ (145 bp product); Ikap20for 5′-GAGGTTGTTCATCATCGGGCC-3′ and Ikap23rev 5′-GGAGGGTACATGGTCTTTGTGA-3′ (245 bp product); Ikap18for and Ikap23rev (see above, 475 bp product); Itgβ3for 5′-AGTGTAAGAAGTTCAACCGGGG-3′ and Itgβ3rev 5′-CTTCCAGATGAGCAGAGTAGCA-3′ (300 bp product); Gata5for 5′-AGGCCACTGGCAATGAAAAAGG-3′ and Gata5rev 5′-GGCCAGAGCACACCAGGTCT-3′ (350 bp product); Gata4for 5′-CCATCCAGTGCTGTCTGCTCT-3′ and Gata4rev 5′-ACTTTGCTGGCCCCCACGTC-3′ (205 bp product); Srffor 5′-TGCCCGCCACCATCATGACG-3′ and Srfrev 5′-TCCCAGCTTGCTGCCCTATCA-3′ (290 bp product); Nkx2-5for 5′-TTTTACCCGGGAGCCTACGGT-3′ and Nkx2-5rev 5′-CCGCTGTTGCTTGAAGCGCC-3′ (210 bp product); Smad4for 5′-ATCCTTCGGGAGGAGATCGCT-3′ and Smad4rev 5′-CGCCTGTTGCTGCATCTGCC-3′ (290 bp product); Bmp4for 5′- CTGGGGAGGAGGAGGAGGAA-3′ and Bmp4rev 5′- TGCTCCCGAAAGAGCCGGAG-3′ (220 bp product); Pai1for 5′- CCCGCCTCCTCATCCTGCC-3′ and Pai1rev 5′- TCGGGTTGTGCCGAACCACAA-3′ (290 bp product); Id1for 5′- GCAGCAGGTGAACGTCCTGC-3′ and Id1rev 5′- ATGCGATCGTCGGCTGGAACA-3′ (280 bp product).

For internal controls, β-actin mRNA or 18S rRNA were amplified by PCR with 22 cycles consisting of 1 min denaturation at 94°C, and 2 min annealing and extension at 72°C, or 17 cycles consisting of 45 sec denaturation at 94°C, 45 sec annealing at 61°C, and 1 min extension at 72°C, respectively.

The primers used were: β-actinfor 5′-GACAACGGCTCCGGCATGTGCAAAG-3′, and β-actinrev 5′-TTCACGGTTGGCCTTAGGGTTCAGGG-3′ which amplify a 320 bp product, and 18Sfor 5′-GGTGGTGGTGCATGGCCGTT-3′and 18Srev 5′- GCAGCCCCGGACATCTAAGG -3′ which amplify a 200 bp product.

In all cases, one third of the PCR reactions were fractionated in 2% agarose gels.
